# Sesame Meal, Vitamin E and Selenium Influence Goats’ Antioxidant Status

**DOI:** 10.3390/antiox10030392

**Published:** 2021-03-05

**Authors:** Eleni Tsiplakou, Christina Mitsiopoulou, Chrysoula Karaiskou, Marica Simoni, Athanasios C. Pappas, Federico Righi, Kyriaki Sotirakoglou, Nikolaos E. Labrou

**Affiliations:** 1 Laboratory of Nutritional Physiology and Feeding, Department of Animal Science, Agricultural University of Athens, Iera Odos 75, GR-11855 Athens, Greece; chr_mitsiopoulou28@hotmail.com (C.M.); chrysoula89@gmail.com (C.K.); apappas@aua.gr (A.C.P.); 2 Department of Veterinary Science, University of Parma, Via del Taglio, 10, 43126 Parma, Italy; marica.simoni@unipr.it (M.S.); federico.righi@unipr.it (F.R.); 3 Laboratory of Mathematics and Statistics, Department of Natural Resources Management and Agricultural Engineering, Agricultural University of Athens, 11855 Athens, Greece; sotirakoglou@aua.gr; 4 Laboratory of Enzyme Technology, Department of Biotechnology, School of Food, Biotechnology and Development, Agricultural University of Athens, Iera Odos 75, GR-11855 Athens, Greece; lambrou@aua.gr

**Keywords:** oxidative stress, sesame meal, selenium, vitamin E, milk, plasma, goats

## Abstract

This study aimed to determine the impact of sesame meal, selenium (Se), and vitamin E (VitE) on goats’ oxidative status. Thirty mid-lactation crossbred goats were divided into five homogeneous groups, and were fed 1 kg of alfalfa hay and 1.2 kg of concentrates daily. The control group (C) received a basal diet. In the concentrates of the treated groups, 10% of the soybean meal was replaced by sesame meal and no extra VitE or Se (SM), or an extra 60 mg of VitE (SME), or 0.1 mg organic Se (SMSe), or their combination (60 mg VitE and 0,1 mg organic Se/kg of concentrate (SMESe). In the plasma of the goats, the dietary treatments did not affect glutathione reductase, glutathione peroxidase, glutathione transferase, catalase, superoxide dismutase activities, malondialdehyde (MDA) content, or the total antioxidant capacity. A reduction and a trend for lower protein carbonyls content was found in goats fed SM (*p* = 0.03) and SME (*p* = 0.06) compared to SMESe. In the milk, the lactoperoxidase activity decreased with SMSe and SMESe. A numerical decrease in the total antioxidant capacity and an increase in the MDA content in the milk of the SMESe group compared with the other treated groups was found. In mid-lactation goats, SM improves the oxidative status of both the organism and the milk.

## 1. Introduction

Selenium (Se) and vitamin E (VitE) are natural essential micronutrients of milk, which have antioxidant properties [1,2]. Their content in milk is closely related with the type and the origin of the animals’ feedstuffs [3,4], but because—in most cases—the basal diet doesn’t fulfill the animals’ Se and VitE requirements, their synthetic or organic (in the case of Se) forms are usually incorporated in their rations. Both Se and VitE are the most well established dietary supplements, and a number of studies have determined their impact on the oxidative status and oxidative stability of milk by using supplementary doses exceeding the animals’ nutritional requirements [5,6]. More specifically, the oxidative deterioration of stored milk was avoided when red clover-fed cows were supplemented with 1182 mg VitE/animal daily [7]. A reduction of the thiobarbituric acid reactive substances (TBARS) content of cows’ stored milk has been found when their diets were supplemented with 1 g [8] or 8000 IU [9] VitE/animal/day, respectively. The daily dietary supplementation of cows with 25 mg Se/animal didn’t affect the lipid and protein oxidation products, or the radical scavenging activity of their milk when it was exposed to Cu ions or to fluorescent light [5]. No effect on the oxidative stability of cows’ milk has been observed when the animals’ diets were supplemented with Se [10,11]. On the other hand, dietary supplementation with 1000 IU VitE and 0.4 mg Se/day simultaneously improved the plasma antioxidant status and reduced the milk fat oxidation of ewes, as found recently by Pulido et al. [6], indicating a synergistic role between these compounds (Se and VitE).

However, lately, natural antioxidants have gained consumers’ interest because they are not toxic and have fewer potential hazards for their health than synthetic antioxidants [12,13]. Moreover, natural antioxidants might also be able to protect both ruminant organisms and milk from oxidative stress, especially when their nutritional requirements are not particularly high. Besides this, the same vitamin status (provitamin A and vitamin E) in both the blood and milk of organic-fed cows have been observed when their diets were supplemented with or without synthetic vitamins, except at the time around parturition [14]. These findings support the idea that synthetic antioxidants might be replaced by natural ones.

Lignans belongs to the natural antioxidant family, and their impact on the antioxidant enzymes’ activities, and on the oxidative stress indicators of blood plasma and milk have been determined in flax meal-fed cows [15]. However, the lignans of sesame (sesaminol, sesamin, sesamol and sesamolin) have a different chemical structure and strong antioxidant index [16,17] but, to the best of our knowledge, their role in the antioxidant capacity of ruminant organisms and milk have not been studied so far.

There are no comparative studies between natural and synthetic antioxidants on the oxidative status of both goat organisms and milk, while at the same time, there are no updated established inclusion levels for both Se and VitE which are suggested to protect animals from oxidative stress and milk from autoxidation. This work was part of a project designed to assess the impact of the dietary inclusion of sesame meal, Se, and VitE on goat’s milk chemical composition and antioxidant status. The influence of dietary sesame meal, VitE, and Se supplementation on goat’s milk production, composition, and fatty acid profile was previously assessed [18].

The aim of the present study was to determine the impact of sesame meal alone (SM) and/or combined with VitE or Se, or VitE and Se simultaneously on both the animal and milk’s oxidative status.

## 2. Materials and Methods

### 2.1. Animals and Diets

Thirty mid-lactation (90 ± 5 days in milk) crossbred dairy goats (Alpine x Local (Greek) breed) of comparable age (3 to 4 years old) were maintained in the Department of Nutritional Physiology and Feeding of the Agricultural University of Athens. The study was conducted according to directive 2010/63/EC on the protection of animals used for scientific purposes, and was approved by the Ethical Committee of the Faculty of Animal Science (019/10032015) of the Agricultural University of Athens. After a two week adaptation period, the animals were divided into five homogeneous sub-groups (n = 6) balanced by their mean body weight (46.5 ± 1.48 kg) and fat-corrected milk yield (1.8 ± 0.11 kg). All of the animals were fed, on a group basis, according to their average energy and crude protein requirements (maintenance plus lactation) with 1 kg of alfalfa hay and 1.2 kg of concentrates, with a forage/concentrate ratio 46/54 [19]. The animals were fed on a group basis, considering their average energy and nutritional requirements, in order for the experimental design to represent the typical commercial farm feeding management, and in order for the results to have practical implications for small ruminants. Parturitions in small ruminants are, in fact, gathered in a very short interval, which means that the animals have similar requirements, and consequently feed intakes. Thus, in this trail, based on the animals’ nutritional requirements, the consumption of both alfalfa hay and concentrates was modified by less than 5% throughout the experimental period, which lasted 42 days. Two types of concentrates were used in this study; one for the control (C) and another for the four treated groups. In order for these two types of concentrates to be iso-energetic and iso-proteic, a partial substitution of soybean by sesame meal (SM) was performed at the level of 10%. This amount was chosen based on findings concerning the dietary inclusion of flax meal, which has similar antioxidant compounds, such as lignans [15]. In both types of concentrates (for the control (C) and for the four treatments) the same vitamins and trace minerals premix was added, containing the following (per kg as mixed): 0.8 mg Co, 3.5 mg I, 70 mg Fe, 90 mg Mn, 100 mg Zn, 125 mg Mg, 0.1 mg organic Se (Se yeast), 3 mg biotin, 14,000 IU vitamin A (retinol), 3500 IU vitamin D (cholecalciferol) and 60 mg vitamin E (90% α-tocopherol). However, in the four sesame meal-treated groups, further from those quantities which were already included in the vitamins and trace minerals premix, either no extra VitE and Se were added (SM), or an extra 60 mg VitE/kg of concentrates (SME), or 0.1 mg organic Se/kg of concentrates (SMSe), or their combination (60 mg VitE and 0.1 mg organic Se/kg of concentrates (SMESe)) were incorporated into the concentrates. The additional levels were decided considering that the organic Se should not exceed 0.2 mg per kg of complete feed [20], and the additional levels of VitE were calculated in order to be close to the goats’ requirements [19], especially with the highest inclusion level. The remaining requirements for antioxidants were considered to be covered by the sesame meal lignans. The total antioxidant content was verified among the dietary treatments by using the Ferric Reducing Ability Power (FRAP) assay, and it gave similar values (Table 1). The inclusion level of sesame meal was decided based on the economical sustainability of the soybean meal substitution as a protein source. The two types of concentrates with the specific vitamin and trace minerals premix for each dietary treatment were provided by the DSM -Nutritional Products Hellas company (Athens, Greece).

The alfalfa hay and concentrates’ chemical compositions are provided in Table 1, together with the estimation of the whole diet’s composition and the daily nutrients intake, which was performed through the NDS software version 3.9.8.04 (Rumen S.a.s. Reggio Emilia, Italy) by using the model for small ruminants [21]. The sesame meal had a high protein (50.1%) and lipid (20%) content. The alfalfa hay was provided separately from the concentrates, and they were both offered to the animals twice a day (in two equal parts at 08:00 and 18:30 h) after milking. The available feeding space was higher than that recommended for adult housed ewes [22] considering the more aggressive behavior of goats [23], in order to favor simultaneous access and lower competitive interactions at the feeder among animals. Thus, diet selectivity did not occur, and no refusals of forage and/or concentrates were observed.

### 2.2. Sample Collection

Individual blood samples were taken at the 14th, 28th and 42nd experimental day, from the jugular vein into 17 Units/mL heparine-containing tubes (BD Vacutainer, Plymouth, UK) for the enzymes, antioxidant capacity, and oxidative stress indicator determination. After collection, the blood samples were centrifuged at 2700× *g* for 15 min in order to separate the plasma from the cells. Individual milk samples were collected at the beginning of the experiment for the animals’ allocation into groups. Moreover, individual milk samples were taken at the 14th, 28th and 42nd day after the pooling of the 10% milk yield from the evening and the morning milking, respectively, for the determination of the same above parameters. Both the milk and blood plasma samples were stored at −80 °C for further analysis.

### 2.3. Samples Analyses

#### 2.3.1. Chemical Composition and Se Determination of the Feed Samples

Individual samples from the alfalfa hay and concentrates were taken at the beginning of the experiment. The feed samples were analyzed for organic matter (OM; official method 7.009), dry matter (DM; official method 7.007) crude protein (CP; official method 7.016) and ether extract (EE; official method 7.060) according to the Association of Official Analytical Chemists [24], and for neutral detergent fibre (NDF), assayed without a heat-stable amylase, and acid detergent fibre (ADF), expressed exclusive of residual ash, according to Van Soest et al. [25]. The selenium was determined in the feed samples using inductively-coupled plasma mass spectrometry (ICP-MS) (Perkin Elmer, Elan 9000, Perkin Elmer Life and Analytical Sciences Inc, Waltham, MA, USA), as described in a previous work [26].

#### 2.3.2. Antioxidant Content Determination of the Dietary Treatments

##### Extract Preparation of the Concentrates

The total antioxidant capacity determination was performed by Ferric Reducing Antioxidant Power (FRAP) assay in quadruplicate, according to Benzie and Strain [27], and the results were expressed as μΜ ascorbic acid/1g dry matter.

#### 2.3.3. Blood Plasma and Milk Analyses

The activities of glutathione reductase (GR), glutathione peroxidase (GPx), glutathione transferase (GST), catalase (CAT), and superoxide dismutase (SOD) were determined in the blood plasma, along with the activities of SOD, GR and lactoperoxidase (LPO) in the goats’ milk. Furthermore, the oxidative stress indicators of the total antioxidant and free radical scavenging activity (Ferric Reducing Ability of Plasma (FRAP) and 2,2’-azino-bis (3-ethylbenzothiazoline-6-sulphonic acid) (ABTS) assays) and oxidative stress biomarkers (malondialdehyde (MDA) and protein carbonyls (PC)), were also determined in the blood plasma and milk of the animals. The enzyme activities, oxidative stress biomarkers, and total antioxidant capacity were measured spectrophotometrically (Helios alpha, UNICAM, Cambridge, UK), as previously described by Tsiplakou et al. [28].

### 2.4. Statistical Analyses

The experimental data were analyzed using the SPSS statistical package (Version 20.0. Armonk, NY: IBM Corp.). The enzyme activities, lipid peroxidation, protein carbonyls, and antioxidant capacity were analyzed using a general linear model (GLM) for repeated measures, considering the sampling time (T) as the repeated measure, with fixed effects of dietary treatment (D) (C, SM, SME, SMSe, SMESe), sampling time (T) (14th, 28th, 42th experimental day), and the interactions among them (D×T) according to the model:

ϒijk = μ + Di + Tj + (D × T) ij +Ak+ eijk

where Υijk is the dependent variable, μ is the overall mean, Di is the effect of dietary treatment (i = 1, 2,...5), Tj the effect of sampling time(j = 1, 2, 3), (D × T) ij the interaction between dietary treatments and sampling time, Ak the animal’s random effect, and eijk is the residual error. Post hoc analyses were performed, when appropriate, using Duncan’s multiple range test, and significance was set at 0.05.

## 3. Results

### 3.1. Blood Plasma

The dietary inclusion of sesame meal alone or in combination with VitE, or Se or VitE and Se simultaneously, didn’t affect the enzyme activities or the total antioxidant capacity in the goats’ blood plasma (Table 2). Significantly higher PC content in the blood plasma of SMESe-fed goats compared with the SM and SME groups was found. Moreover, sesame meal alone, in comparison with VitE (SME) or Se (SMSe) simultaneously, or even when it was not included in the concentrate (C), resulted the lowest values in PC content in the goats’ plasma without the results being significant. The sampling time affected all of the parameters in the blood plasma of goats. More specifically, the lowest GR and GST activities were observed at the 28th experimental day. The same trend was found for the GPx and CAT activities, as well as for both the MDA and PC content at the 28th day compared with 14th experimental day. The ABTS values were similar until the 28th experimental day, but decreased at the 42nd day of the trial. At the 42nd experimental day, all of the other values were either similar (GPx, CAT, SOD, MDA) or higher (GR, GST, PCs) than the previous sampling day. There was a significant interaction between diet and sampling time for FRAP (*p* < 0.05) and ABTS (*p* < 0.01) (Table 2).

### 3.2. Milk

A significant reduction of the LPO activity in the milk of the SME- and SMESe-fed goats compared with the C group was observed (Table 3). The lowest and the highest values of FRAP and MDA, respectively, were observed in the milk of the SMESe-fed goats compared with the other treated groups, with the results being significant for the MDA content between the SMESe and SME treatments. The SOD activity, the MDA and PC content, and the total antioxidant capacity measured by the FRAP assay declined significantly in the goats’ milk throughout the sampling period. No significant interactions between diet and sampling time were found.

## 4. Discussion

### 4.1. Blood Plasma

Similarly to our results, no differences of the GPx, CAT and SOD activities in cows’ blood plasma were found when the animals were fed with 5, 10, or 15% flax meal [15]. Moreover, the dietary inclusion with 16% flax meal didn’t change the GPx and SOD activities in cows’ blood plasma [29]. On the contrary, in cows, Lima et al. [30] observed a significant decrease and a trend for increase in the CAT activity in blood plasma and erythrocytes, respectively, when the animals were fed a diet containing 12.4% flax meal. Both sesame and flax seeds are rich in plant lignans [31], which are strong antioxidant compounds [32]. Moreover, not only lignans by themselves but also other molecules which are produced by their metabolism in the rumen [33] end up in the animals’ blood [34], such as enterolactones [35], and also have strong antioxidant effect.

However, in the present study, the natural antioxidants (sesame meal), alone or in combination with the synthetic ones (VitE and Se), didn’t affect the MDA content and the total antioxidant capacity, as determined by the use of the FRAP and ABTS assays, in goats’ blood plasma. Similarly, the dietary supplementation with flax meal had no effect on the peroxidability index [36] and TBARS production in cows’ blood plasma [15,36]. The inability of plant lignans, alone or together with VitE, or Se alone or in combination, to affect both the oxidative status and the oxidative stress indicators in most of the aforementioned studies might be due to the fact that the animals which were used in these experiments were at the mid-lactation stage, and consequently were not exposed to oxidative stress. Thus, it would be interesting if these antioxidants compounds could be evaluated in goats under stress conditions, such as the high fat diets which cause milk fat depression, because they might have a completely different response. Indeed, it has been proven that cows fed with a high fat diet supplemented with a high quantity of Se (0.3 mg/kg DM) had significantly higher blood plasma GPx activity [37].

A trend for higher PC content in the blood plasma of goats fed with SMESe, compared with those that consumed the SME diet (*p* = 0.06), was found (data not shown). On the other hand, it should be pointed out that a significantly lower PC content in the blood plasma of the SM fed goats’, compared with those that consumed the SMESe diet, was observed (*p* = 0.03; Table 2). These findings might indicate that the natural antioxidants of sesame meal had the best impact on the antioxidant capacity of goats. These results might also indicate that the dietary supplementation with high levels of antioxidant compounds might negatively affect the antioxidant capacity of the goats’ organism, because proteins are the most oxidative damage-susceptible molecules in cells [38,39]. Indeed, it has been shown, in humans, that the over consumption of antioxidants, especially synthetic ones, leads to a situation known as ‘antioxidative stress’, with harmful effects in the organism [40,41]. Moreover, the dietary supplementation of cows with Se and VitE simultaneously, at both low (0.15 mg/kg DM and 5000 IU/day/animal) and high (0.3 mg/kg DM and 10,000 IU/day/animal) levels, significantly enhanced their blood plasma GPx activity compared with those fed a high fat-basal diet, alone or in combination with Se (0.15 mg/kg DM) or vitamin E (5000 IU/day/animal) [37]. Additionally, Liu et al. [37] observed a reduction in TBARS production in cows’ blood plasma when a high fat-basal diet, supplemented simultaneously with Se and VitE at either low (0.15 mg/kg DM and 5000 IU/day/animal) or high (0.3 mg/kg DM and 10,000 IU/day/animal) levels in relation with the un-supplemented ones, or those supplemented with VitE (5000 or IU/day/animal) or Se (0.15 or 0.30 mg/kg DM) separately, indicating that the high consumption of antioxidants may enhance oxidative stress. Moreover, it should be underlined here, that not only the amount of antioxidants in the animals’ diet, but also their mode of action, seems to have different responses in organism’s oxidative status. Additionally, the duration of the antioxidant consumption seems to affect differently the organism’s oxidative status in terms of enzyme activities, oxidative stress indicators, and antioxidant capacity measurements. Particularly, it seems that the 28 days compared to 42 days of antioxidant consumption favors the oxidative capacity of goats, indicating that long term antioxidant consumption needs further research, because it might lead to oxidative stress.

### 4.2. Milk

A significant reduction of the LPO activity in the milk of SME-and SMESe-fed goats compared with the control group was observed.

The strong inhibitory effects of α-tocopherol on LPO activity has been proven recently in bovine milk in vitro [42]. On the other hand, the natural antioxidants of sesame meal didn’t affect negatively the LPO activity in goats’ milk (Table 3). This is very important because this enzyme has anti-microbial effects which erase the harmful effects of microorganisms in the animals’ mammary glands [43]. Moreover, the LPO system has a pivotal role in dairy industry in the preservation of raw milk [44], cheese [45], and yogurt [46], and can be used to extend the shelf-life of pasteurized milk as well [47,48].

Moreover, it should be pointed out that the dietary supplementation of the two synthetic antioxidants (VitE and Se) simultaneously, not only caused the most intense decline in the LPO activity in goat’s milk, but also resulted in the lowest FRAP and the highest MDA values in their milk compared with those derived from the other treated animals. This might show, again, that the dietary supplementation with high levels of antioxidants might have a negative impact on the oxidative status of goats’ milk, as was previously discussed for the goats’ organism (blood plasma). Indeed, Schogor et al. [15] observed that 5 and 10%, compared to 0 and 15% of dietary flax meal can improve the oxidative status (decreased TBARS–MDA equivalents) in cows’ milk. Dietary supplementation with sesame meal alone (SM) or in combination with Se (SMSe) or VitE (SME), or Se and VitE simultaneously (SMESe), didn’t modify the GR and SOD activities in goats’ milk. This might indicate a sufficient pool of non-enzymatic antioxidant substrates in goats’ milk. Indeed, an increase in the total milk antioxidant capacity (measured with the FRAP assay) of treated goats in comparison with the C group was found, which strengthens this assumption, although the SMESe diet results were significant for those fed.

Finally, the SOD activity reduction throughout the sampling period has also been previously documented in goat [49] and human milk [50], as the lactation period is proceeded. As mentioned for blood plasma, the duration of the antioxidant consumption might affect also the milk’s oxidative stability. Unlike the blood plasma findings, the long term (42 vs. 28 and 14 days respectively) antioxidant consumption improves goats’ milk’s oxidative capacity.

## 5. Conclusions

The incorporation of sesame meal at 10% in goats’ diets, at mid-lactation, could be a good nutritional strategy to reduce not only the oxidative stress but also to preserve the oxidative stability of milk. Unexpectedly, the combination of the tested antioxidant appeared to enhance the oxidative stress markers in both the goats’ organism and milk. The duration of the administration of the tested antioxidants at these specific amounts appeared to affect the oxidative status of the organism and milk in an opposite fashion. Thus, more research is needed to define their proper inclusion in lactating goats’ diets.

## Figures and Tables

**Table 1:** Components, calculated composition, and Se and vitE concentrations of the diets administered to the groups of dairy goats involved in the trial.

		Diets/Groups				
		C ^1^	SM ^2^	SME ^3^	SMSe ^4^	SMESe ^5^
Item ^6^						
Diet components (%DM)						
Mixed hay ^7^		46.35	46.15	46.15	46.15	46.15
Barley meal		18.88	18.80	18.80	18.80	18.80
Cracked maize meal		15.81	15.74	15.74	15.74	15.74
Wheat meal		5.26	6.29	6.29	6.29	6.29
Sesame meal		-	5.85	5.85	5.85	5.85
Soybean meal		11.91	5.39	5.39	5.39	5.39
Calcium		1.35	1.35	1.35	1.35	1.35
Sodium Chloride		0.32	0.32	0.32	0.32	0.32
Trace Mineral Vitamin Premix ^8^		0.12	0.12	0.12	0.12	0.12
Calculated composition (% DM)						
Daily feed intake (g) (in DM basis)		1934	1878	1902	1923	1872
Ash		7.75	7.94	7.94	7.94	7.94
aNDFom		28.28	28.72	28.72	28.72	28.72
ADF		16.32	16.35	16.35	16.35	16.35
ADL		3.85	3.93	3.93	3.93	3.93
NFC		45.21	43.31	43.31	43.31	43.31
Starch		24.14	23.98	23.98	23.98	23.98
Sugars		7.06	6.57	6.57	6.57	6.57
EE		2.34	3.45	3.45	3.45	3.45
CP		16.40	16.60	16.60	16.60	16.60
Sol CP		4.83	5.25	5.25	5.25	5.25
RDP3x		10.74	10.96	10.96	10.96	10.96
ENl (Mcal/kg)		1.6	1.6	1.6	1.6	1.6
Se (mg/kg)	Analysed	0.192	0.185	0.170	0.294	0.290
	Calculated	0.210	0.220	0.220	0.280	0.282
VitE (mg/kg)	Analysed	60.80	60.82	99.32	60.82	99.34
	Calculated	76.41	76.24	112.38	76.24	112.18
Antioxidant content (FRAP) (μΜ ascorbic acid/g DM)		7032	9047	8176	9234	8671

^1^ C = control, ^2^ SM = sesame meal, ^3^ SME = SM + 60 mg of VitE/kg concentrate, ^4^ SMSe = SM + 0.1 mg organic Se/kg concentrate, ^5^ SMESe = SM + 60 mg of VitE + 0.1 mg organic Se/kg concentrate, ^6^ DM: dry matter; aNDFom: amylase treated NDF ash free; ADF: acid detergent fibre; ADL: acid detergent lignin; NFC: non-fibrous carbohydrates; EE: ether extracts; CP: crude protein; Sol CP: soluble protein; RDP3x: rumen degradable protein (3 × maintenance); ENl: net energy for lactation; Se: Selenium; VitE: vitamin E; ^7^ mixed hay: DM 92.47%; Ash 7.96%; aNDFom 38.90%; CP 13.42%; EE 1.54%, ^8^ trace mineral vitamin premix of C (as mixed): DM 96.59%, ash 88.86%, Mg 62,500 mg/kg, Mn 45,000 ppm, Fe 35,000 ppm, Zn 50,000 ppm, I 1.750 ppm, Co 400 ppm, Se 50 ppm, Vit A 7,000,000 UI/kg, Vit D3 1,750,000 UI/kg, Vit E 30,000 mg/kg, Biotin 1.500 mg/kg. Trace mineral vitamin premix of SM—(see Control description). Trace mineral vitamin premix of SME—(see Control description) + Vit E 30,000 mg/kg. Trace mineral vitamin premix of SMSe + Se 50 ppm. Trace mineral vitamin premix of SMESe + Vit E 30,000 mg/kg + Se 50 ppm.

**Table 2:** Enzyme activities (Units/mL), total antioxidant capacity, and oxidative stress biomarkers in the blood plasma of goats fed the five diets at the three sampling times.eldar tactics.

	Diet (D)						Sampling Day(T)				Effect		
	C ^1^	SM ^2^	SME ^3^	SMSe ^4^	SMΕSe ^5^	SEM ^15^	14	28	42	SEM ^15^	D	T	DxT
GR ^6^	0.05	0.06	0.06	0.06	0.06	0.004	0.06 ^A^	0.05 ^B^	0.06 ^A^	0.002	NS	*	NS
GPx ^7^	2.48	2.73	2.81	2.79	2.60	0.200	2.88 ^A^	2.51 ^B^	2.65 ^A,B^	0.132	NS	*	NS
GST ^8^	0.13	0.14	0.13	0.13	0.13	0.007	0.15 ^A^	0.11 ^B^	0.14 ^A^	0.005	NS	***	NS
CAT ^9^	127.75	120.61	114.04	131.30	100.29	13.728	133.56 ^A^	104.92 ^B^	117.92 ^A.B^	7.056	NS	***	NS
SOD ^10^	16.31	16.54	16.34	16.46	15.81	0.512	15.90 ^A^	16.49 ^A,B^	16.49 ^B^	0.274	NS	*	NS
FRAP ^11^	1.29	1.30	1.41	1.43	1.25	0.069	1.32	1.31	1.37	0.036	NS	*	*
ABTS ^12^	34.71	33.82	33.91	34.76	32.89	0.724	35.43 ^A^	35.16 ^A^	31.48 ^B^	0.403	NS	***	**
MDA ^13^	0.70	0.67	0.66	0.65	0.66	0.020	0.72 ^A^	0.66 ^B^	0.63 ^B^	0.017	NS	*	NS
PCs ^14^	2.28 ^a,b,c^	2.12 ^c,b^	2.20 ^c^	2.31 ^a,b,c^	2.68 ^a^	0.174	2.36 ^A^	1.76 ^C^	2.83 ^B^	0.126	*	***	NS

Means with different superscripts in each row among the dietary treatments (a, b, c) and the three-sampling times (A, B, C) for each parameter differ significantly (*p* ≤ 0.05). ^1^ C = control; ^2^ SM = sesame meal; ^3^ SME = sesame meal +60 mg of VitE/kg concentrate; ^4^ SMSe = sesame meal + 0.1 mg organic Se/kg concentrate; ^5^ SMESe = sesame meal + 60 mg of VitE + 0.1 mg organic Se/kg concentrate; ^6^ GR: glutathione reductase; ^7^ GPx: glutathione peroxidase; ^8^ GST: glutathione transferase; ^9^ CAT: catalase; ^10^ SOD: superoxide dismutase; ^11^ FRAP: ferric reducing ability of plasma (μΜ ascorbic acid); ^12^ ABTS: 2,2’-azino-di (3-ethylbenzthiazoline-6-sulfonic acid (inhibition %); ^13^ MDA: malondialdehyde (μΜ); ^14^ PCs: protein carbonyls (nmol/mL); ^15^ SEM = standard error of the mean; * = *p* < 0.05, ** = *p* < 0.01, *** = *p* < 0.001.

**Table 3:** Enzyme activities (Units/mL), total antioxidant capacity, and oxidative stress biomarkers in the milk of goats fed the five diets at the three sampling times.

	Diet (D)						Sampling Time ^13^(T)				Effect		
	C ^1^	SM ^2^	SME ^3^	SMSe ^4^	SMΕSe ^5^	SEM ^14^	14	28	42	SEM^14^	D	T	DxT
GR ^6^	0.23	0.22	0.15	0.29	0.29	0.030	0.15 ^B^	0.29 ^A^	0.27 ^A^	0.023	NS	**	NS
SOD ^7^	67.60	80.11	78.13	79.63	81.09	5.802	82.39 ^A^	76.31 ^A,B^	73.24 ^B^	3.026	NS	**	NS
LPO ^8^	0.61 ^a^	0.51 ^a,b^	0.47 ^a,b,c^	0.39 ^b,c^	0.35 ^c^	0.052	0.54	0.43	0.43	0.033	*	NS	NS
FRAP ^9^	1.92 ^a^	2.49 ^a,b^	2.62 ^b^	2.57 ^b^	2.17 ^a,b^	0.178	2.36 ^B^	2.61 ^A^	2.09 ^C^	0.103	*	***	NS
ABTS ^10^	44.13	42.88	47.02	44.15	48.84	2.567	43.67	45.98	46.56	1.437	NS	NS	NS
MDA ^11^	0.65 ^a^	0.56 ^a,b^	0.54 ^b^	0.57 ^a,b^	0.63 ^a^	0.028	0.80^A^	0.54 ^B^	0.43 ^C^	0.019	*	***	NS
PCs ^12^	1.72	1.58	1.36	1.61	1.49	0.088	1.84^A^	1.66 ^A^	1.16 ^B^	0.060	NS	***	NS

Means with different superscripts in each row among the dietary treatments (a, b, c) and the three-sampling time (A, B, C) for each parameter differ significantly (*p* ≤ 0.05).^1^ C = control; ^2^ SM = sesame meal; ^3^ SME = sesame meal +60 mg of VitE/kg concentrate; ^4^ SMSe = sesame meal +0.1 mg organic Se/kg concentrate; ^5^ SMESe = sesame meal +60 mg of VitE + 0.1 mg organic Se/kg concentrate; ^6^ GR: glutathione reductase; ^7^ SOD: superoxide dismutase; ^8^ LPO = lactoperoxidase; ^9^ FRAP: ferric reducing ability of plasma (μΜ ascorbic acid); ^10^ ABTS: 2,2’-azino-di (3-ethylbenzthiazoline-6-sulfonic acid (inhibition %); ^11^ MDA: malondialdehyde (μΜ); ^12^ PCs: protein carbonyls (nmol/mL); ^13^ experimental day; ^14^ SEM = standard error of the mean; * = *p* < 0.05, ** = *p* < 0.01, *** = *p* < 0.001.

## Data Availability

Not applicable.
